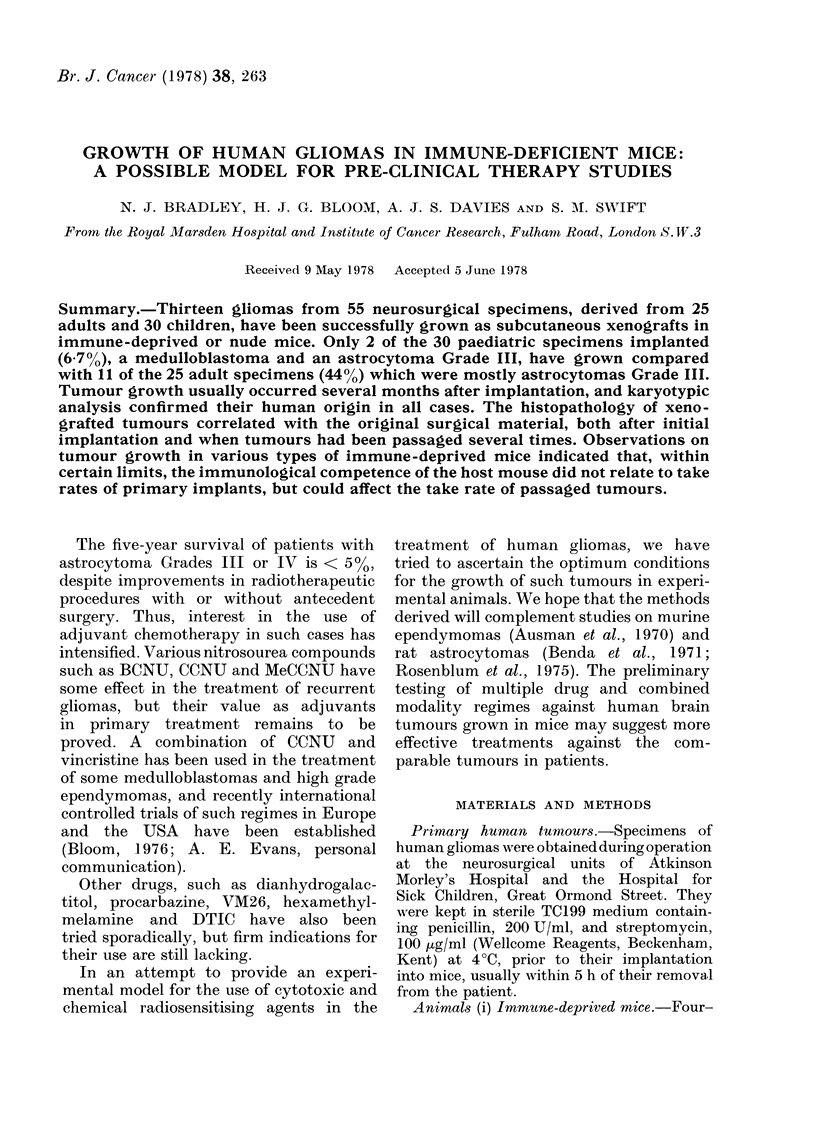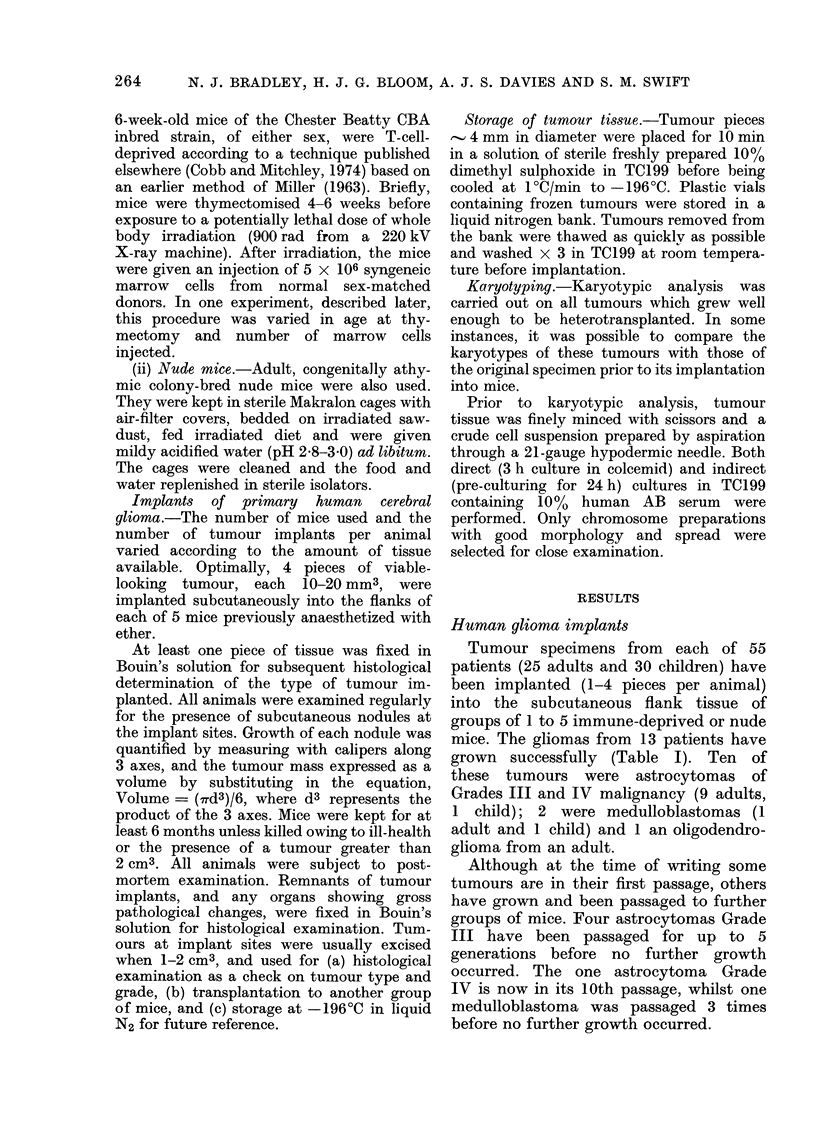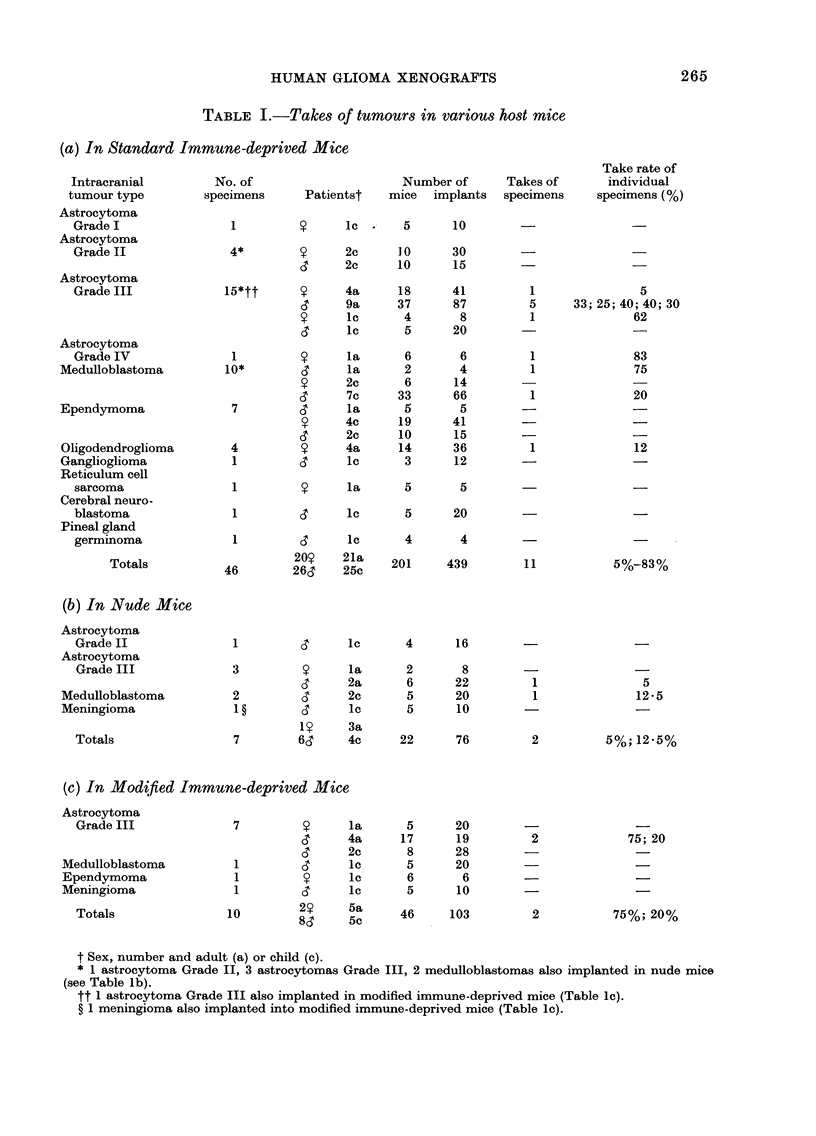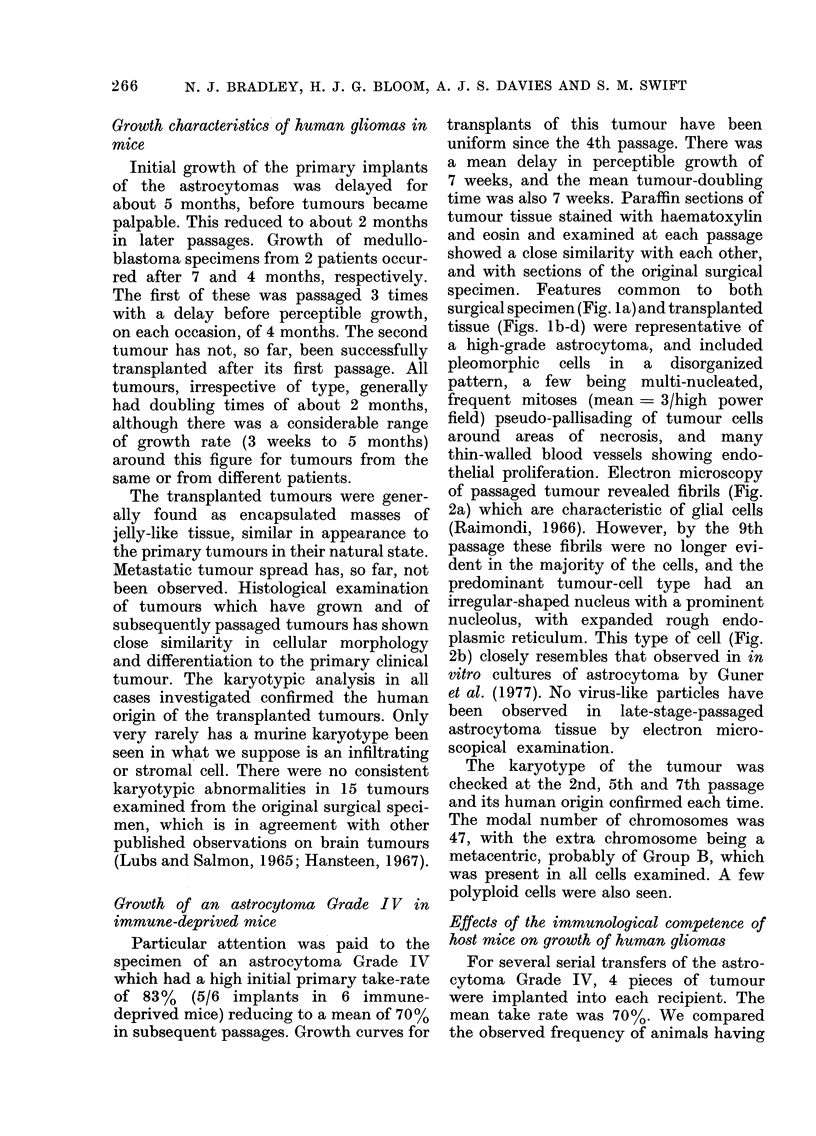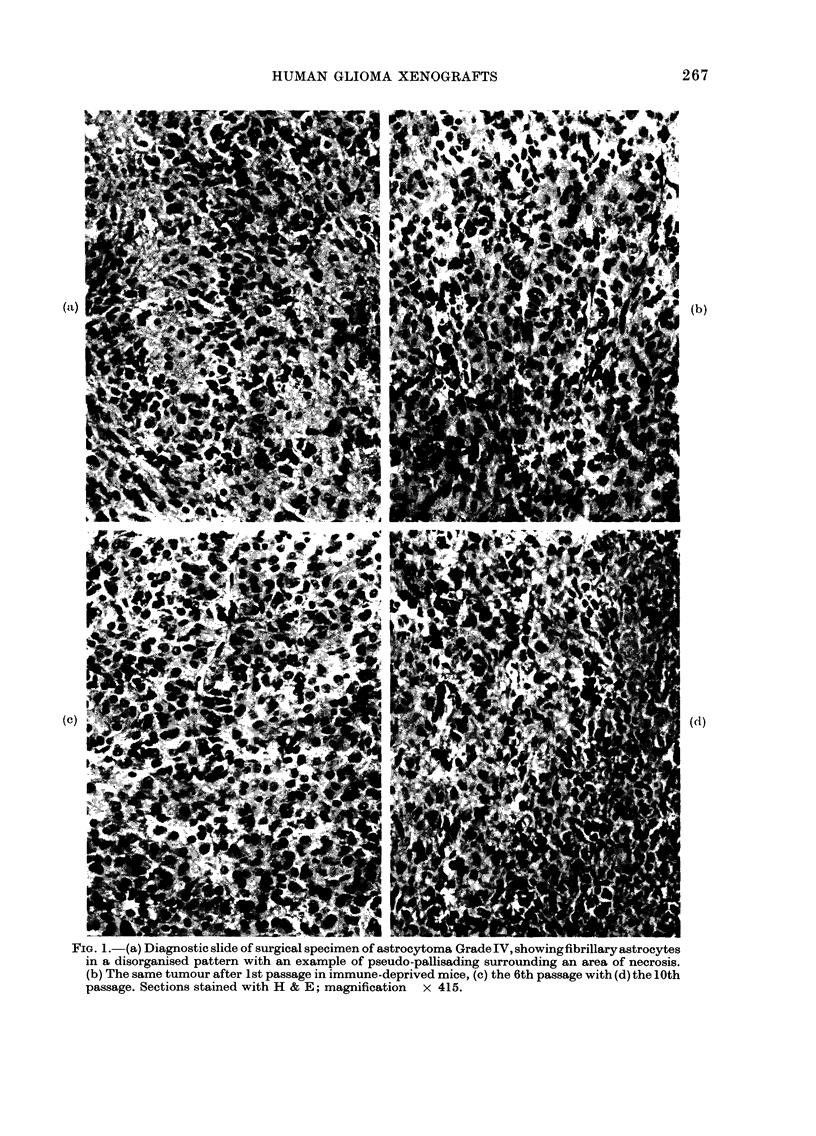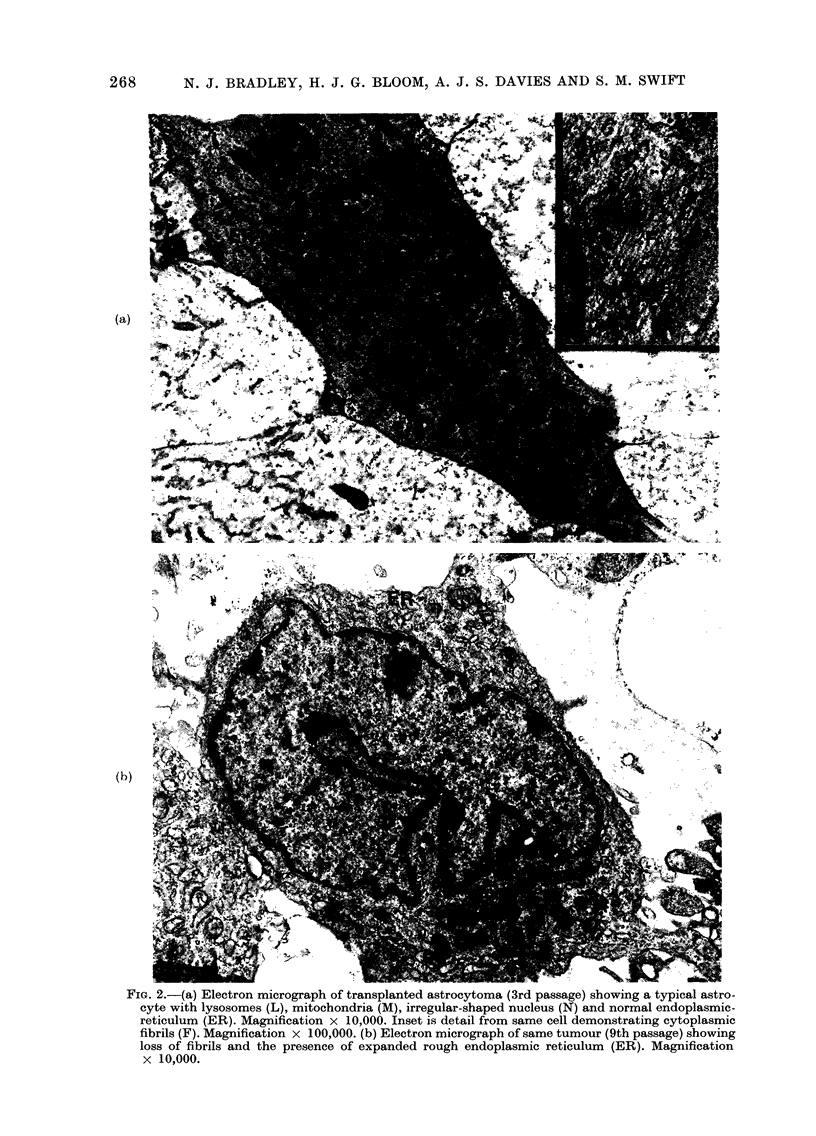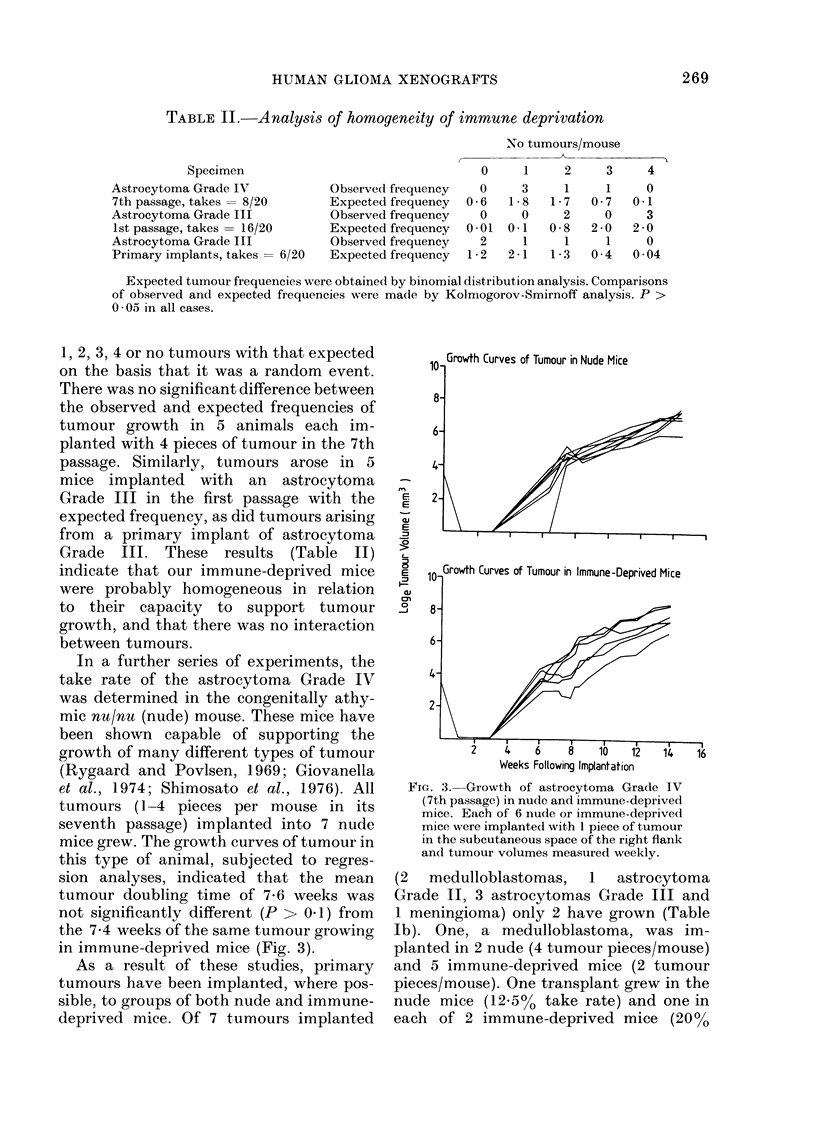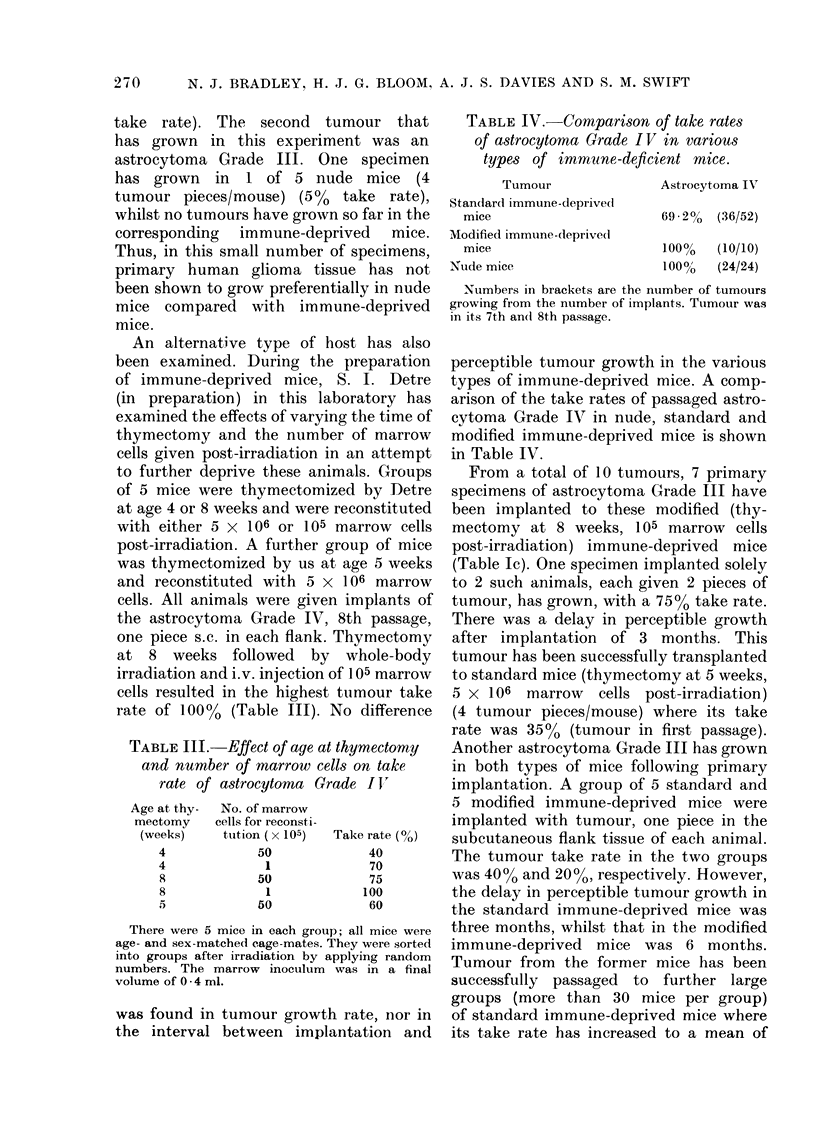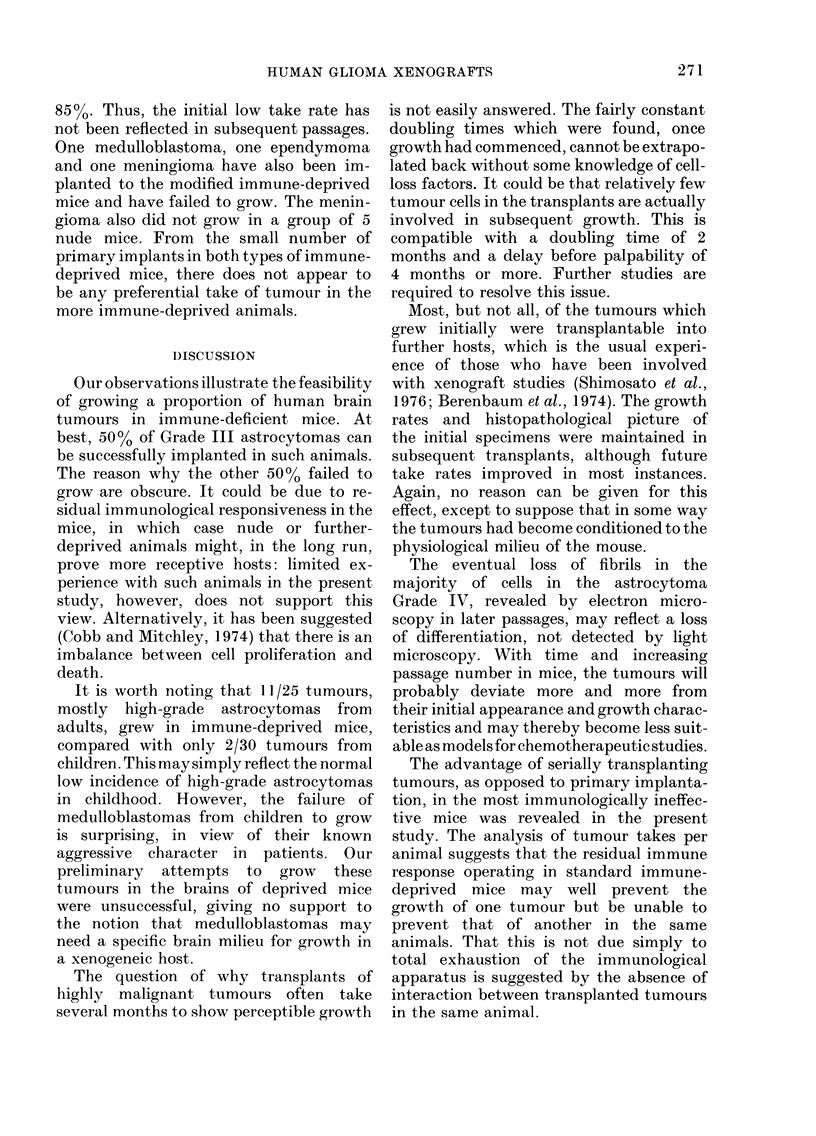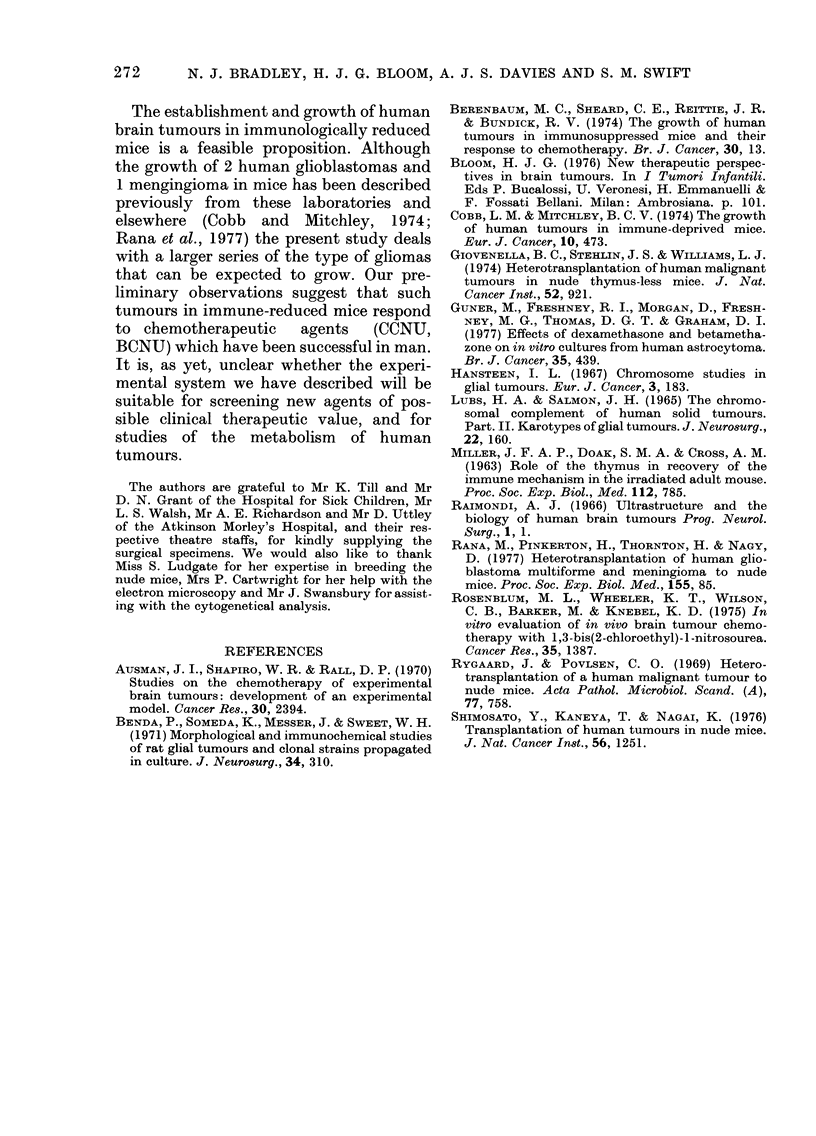# Growth of human gliomas in immune-deficient mice: a possible model for pre-clinical therapy studies.

**DOI:** 10.1038/bjc.1978.197

**Published:** 1978-08

**Authors:** N. J. Bradley, H. J. Bloom, A. J. Davies, S. M. Swift

## Abstract

**Images:**


*Br. J. Cancer* (1978) **38**, 263

### GROWTH OF HUMAN GLIOMAS IN IMMUNE-DEFICIENT MICE: A POSSIBLE MODEL FOR PRE-CLINICAL THERAPY STUDIES

N. J. BRADLEY, H. J. G. BLOOM, A. J. S. DAVIES AND S. M. SWIFT

*From the Royal Marsden Hospital and Institute of Cancer Research, Fulham Road, London S.W.3*

Received 9 May 1978 Accepted 5 June 1978

**Summary.—Thirteen gliomas from 55 neurosurgical specimens, derived from 25 adults and 30 children, have been successfully grown as subcutaneous xenografts in immune-deprived or nude mice. Only 2 of the 30 paediatric specimens implanted (6·7%), a medulloblastoma and an astrocytoma Grade III, have grown compared with 11 of the 25 adult specimens (44%) which were mostly astrocytomas Grade III. Tumour growth usually occurred several months after implantation, and karyotypic analysis confirmed their human origin in all cases. The histopathology of xenografted tumours correlated with the original surgical material, both after initial implantation and when tumours had been passaged several times. Observations on tumour growth in various types of immune-deprived mice indicated that, within certain limits, the immunological competence of the host mouse did not relate to take rates of primary implants, but could affect the take rate of passaged tumours.**

The five-year survival of patients with astrocytoma Grades III or IV is $< 5\%$, despite improvements in radiotherapeutic procedures with or without antecedent surgery. Thus, interest in the use of adjuvant chemotherapy in such cases has intensified. Various nitrosourea compounds such as BCNU, CCNU and MeCCNU have some effect in the treatment of recurrent gliomas, but their value as adjuvants in primary treatment remains to be proved. A combination of CCNU and vincristine has been used in the treatment of some medulloblastomas and high grade ependymomas, and recently international controlled trials of such regimes in Europe and the USA have been established (Bloom, 1976; A. E. Evans, personal communication).

Other drugs, such as dianhydrogalactitol, procarbazine, VM26, hexamethylmelamine and DTIC have also been tried sporadically, but firm indications for their use are still lacking.

In an attempt to provide an experimental model for the use of cytotoxic and chemical radiosensitising agents in the treatment of human gliomas, we have tried to ascertain the optimum conditions for the growth of such tumours in experimental animals. We hope that the methods derived will complement studies on murine ependymomas (Ausman *et al.*, 1970) and rat astrocytomas (Benda *et al.*, 1971; Rosenblum *et al.*, 1975). The preliminary testing of multiple drug and combined modality regimes against human brain tumours grown in mice may suggest more effective treatments against the comparable tumours in patients.

### MATERIALS AND METHODS

*Primary human tumours.*—Specimens of human gliomas were obtained during operation at the neurosurgical units of Atkinson Morley's Hospital and the Hospital for Sick Children, Great Ormond Street. They were kept in sterile TC199 medium containing penicillin, 200 U/ml, and streptomycin, 100 μg/ml (Wellcome Reagents, Beckenham, Kent) at 4°C, prior to their implantation into mice, usually within 5 h of their removal from the patient.

*Animals* (i) *Immune-deprived mice.*—Four-

6-week-old mice of the Chester Beatty CBA inbred strain, of either sex, were T-cell-deprived according to a technique published elsewhere (Cobb and Mitchley, 1974) based on an earlier method of Miller (1963). Briefly, mice were thymectomised 4–6 weeks before exposure to a potentially lethal dose of whole body irradiation (900 rad from a 220 kV X-ray machine). After irradiation, the mice were given an injection of $5 \times 10^6$ syngeneic marrow cells from normal sex-matched donors. In one experiment, described later, this procedure was varied in age at thymectomy and number of marrow cells injected.

(ii) *Nude mice*.—Adult, congenitally athymic colony-bred nude mice were also used. They were kept in sterile Makralon cages with air-filter covers, bedded on irradiated sawdust, fed irradiated diet and were given mildy acidified water (pH 2·8–3·0) *ad libitum*. The cages were cleaned and the food and water replenished in sterile isolators.

*Implants of primary human cerebral glioma*.—The number of mice used and the number of tumour implants per animal varied according to the amount of tissue available. Optimally, 4 pieces of viable-looking tumour, each 10–20 mm³, were implanted subcutaneously into the flanks of each of 5 mice previously anaesthetized with ether.

At least one piece of tissue was fixed in Bouin's solution for subsequent histological determination of the type of tumour implanted. All animals were examined regularly for the presence of subcutaneous nodules at the implant sites. Growth of each nodule was quantified by measuring with calipers along 3 axes, and the tumour mass expressed as a volume by substituting in the equation, Volume = $(\pi d^3)/6$, where $d^3$ represents the product of the 3 axes. Mice were kept for at least 6 months unless killed owing to ill-health or the presence of a tumour greater than 2 cm³. All animals were subject to post-mortem examination. Remnants of tumour implants, and any organs showing gross pathological changes, were fixed in Bouin's solution for histological examination. Tumours at implant sites were usually excised when 1–2 cm³, and used for (a) histological examination as a check on tumour type and grade, (b) transplantation to another group of mice, and (c) storage at −196°C in liquid $N_2$ for future reference.

*Storage of tumour tissue*.—Tumour pieces ~ 4 mm in diameter were placed for 10 min in a solution of sterile freshly prepared 10% dimethyl sulphoxide in TC199 before being cooled at 1°C/min to −196°C. Plastic vials containing frozen tumours were stored in a liquid nitrogen bank. Tumours removed from the bank were thawed as quickly as possible and washed × 3 in TC199 at room temperature before implantation.

*Karyotyping*.—Karyotypic analysis was carried out on all tumours which grew well enough to be heterotransplanted. In some instances, it was possible to compare the karyotypes of these tumours with those of the original specimen prior to its implantation into mice.

Prior to karyotypic analysis, tumour tissue was finely minced with scissors and a crude cell suspension prepared by aspiration through a 21-gauge hypodermic needle. Both direct (3 h culture in colcemid) and indirect (pre-culturing for 24 h) cultures in TC199 containing 10% human AB serum were performed. Only chromosome preparations with good morphology and spread were selected for close examination.

### RESULTS

### *Human glioma implants*

Tumour specimens from each of 55 patients (25 adults and 30 children) have been implanted (1–4 pieces per animal) into the subcutaneous flank tissue of groups of 1 to 5 immune-deprived or nude mice. The gliomas from 13 patients have grown successfully (Table I). Ten of these tumours were astrocytomas of Grades III and IV malignancy (9 adults, 1 child); 2 were medulloblastomas (1 adult and 1 child) and 1 an oligodendroglioma from an adult.

Although at the time of writing some tumours are in their first passage, others have grown and been passaged to further groups of mice. Four astrocytomas Grade III have been passaged for up to 5 generations before no further growth occurred. The one astrocytoma Grade IV is now in its 10th passage, whilst one medulloblastoma was passaged 3 times before no further growth occurred.

TABLE I.—*Takes of tumours in various host mice*

(*a*) *In Standard Immune-deprived Mice*

| Intracranial tumour type | No. of specimens | Patients† | | Number of mice | Number of implants | Takes of specimens | Take rate of individual specimens (%) |
|---|---|---|---|---|---|---|---|
| Astrocytoma Grade I | 1 | ♀ | 1c | 5 | 10 | — | — |
| Astrocytoma Grade II | 4* | ♀ | 2c | 10 | 30 | — | — |
| | | ♂ | 2c | 10 | 15 | — | — |
| Astrocytoma Grade III | 15*†† | ♀ | 4a | 18 | 41 | 1 | 5 |
| | | ♂ | 9a | 37 | 87 | 5 | 33; 25; 40; 40; 30 |
| | | ♀ | 1c | 4 | 8 | 1 | 62 |
| | | ♂ | 1c | 5 | 20 | — | — |
| Astrocytoma Grade IV | 1 | ♀ | 1a | 6 | 6 | 1 | 83 |
| Medulloblastoma | 10* | ♂ | 1a | 2 | 4 | 1 | 75 |
| | | ♀ | 2c | 6 | 14 | — | — |
| | | ♂ | 7c | 33 | 66 | 1 | 20 |
| Ependymoma | 7 | ♂ | 1a | 5 | 5 | — | — |
| | | ♀ | 4c | 19 | 41 | — | — |
| | | ♂ | 2c | 10 | 15 | — | — |
| Oligodendroglioma | 4 | ♀ | 4a | 14 | 36 | 1 | 12 |
| Ganglioglioma | 1 | ♂ | 1c | 3 | 12 | — | — |
| Reticulum cell sarcoma | 1 | ♀ | 1a | 5 | 5 | — | — |
| Cerebral neuroblastoma | 1 | ♂ | 1c | 5 | 20 | — | — |
| Pineal gland germinoma | 1 | ♂ | 1c | 4 | 4 | — | — |
| Totals | 46 | 20♀ 26♂ | 21a 25c | 201 | 439 | 11 | 5%–83% |

(*b*) *In Nude Mice*

| Intracranial tumour type | No. of specimens | Patients† | | Number of mice | Number of implants | Takes of specimens | Take rate of individual specimens (%) |
|---|---|---|---|---|---|---|---|
| Astrocytoma Grade II | 1 | ♂ | 1c | 4 | 16 | — | — |
| Astrocytoma Grade III | 3 | ♀ | 1a | 2 | 8 | — | — |
| | | ♂ | 2a | 6 | 22 | 1 | 5 |
| Medulloblastoma | 2 | ♂ | 2c | 5 | 20 | 1 | 12·5 |
| Meningioma | 1§ | ♂ | 1c | 5 | 10 | — | — |
| Totals | 7 | 1♀ 6♂ | 3a 4c | 22 | 76 | 2 | 5%; 12·5% |

(*c*) *In Modified Immune-deprived Mice*

| Intracranial tumour type | No. of specimens | Patients† | | Number of mice | Number of implants | Takes of specimens | Take rate of individual specimens (%) |
|---|---|---|---|---|---|---|---|
| Astrocytoma Grade III | 7 | ♀ | 1a | 5 | 20 | — | — |
| | | ♂ | 4a | 17 | 19 | 2 | 75; 20 |
| | | ♂ | 2c | 8 | 28 | — | — |
| Medulloblastoma | 1 | ♂ | 1c | 5 | 20 | — | — |
| Ependymoma | 1 | ♀ | 1c | 6 | 6 | — | — |
| Meningioma | 1 | ♂ | 1c | 5 | 10 | — | — |
| Totals | 10 | 2♀ 8♂ | 5a 5c | 46 | 103 | 2 | 75%; 20% |

† Sex, number and adult (a) or child (c).

* 1 astrocytoma Grade II, 3 astrocytomas Grade III, 2 medulloblastomas also implanted in nude mice (see Table 1b).

†† 1 astrocytoma Grade III also implanted in modified immune-deprived mice (Table 1c).

§ 1 meningioma also implanted into modified immune-deprived mice (Table 1c).

*Growth characteristics of human gliomas in mice*

Initial growth of the primary implants of the astrocytomas was delayed for about 5 months, before tumours became palpable. This reduced to about 2 months in later passages. Growth of medulloblastoma specimens from 2 patients occurred after 7 and 4 months, respectively. The first of these was passaged 3 times with a delay before perceptible growth, on each occasion, of 4 months. The second tumour has not, so far, been successfully transplanted after its first passage. All tumours, irrespective of type, generally had doubling times of about 2 months, although there was a considerable range of growth rate (3 weeks to 5 months) around this figure for tumours from the same or from different patients.

The transplanted tumours were generally found as encapsulated masses of jelly-like tissue, similar in appearance to the primary tumours in their natural state. Metastatic tumour spread has, so far, not been observed. Histological examination of tumours which have grown and of subsequently passaged tumours has shown close similarity in cellular morphology and differentiation to the primary clinical tumour. The karyotypic analysis in all cases investigated confirmed the human origin of the transplanted tumours. Only very rarely has a murine karyotype been seen in what we suppose is an infiltrating or stromal cell. There were no consistent karyotypic abnormalities in 15 tumours examined from the original surgical specimen, which is in agreement with other published observations on brain tumours (Lubs and Salmon, 1965; Hansteen, 1967).

*Growth of an astrocytoma Grade IV in immune-deprived mice*

Particular attention was paid to the specimen of an astrocytoma Grade IV which had a high initial primary take-rate of 83% (5/6 implants in 6 immune-deprived mice) reducing to a mean of 70% in subsequent passages. Growth curves for transplants of this tumour have been uniform since the 4th passage. There was a mean delay in perceptible growth of 7 weeks, and the mean tumour-doubling time was also 7 weeks. Paraffin sections of tumour tissue stained with haematoxylin and eosin and examined at each passage showed a close similarity with each other, and with sections of the original surgical specimen. Features common to both surgical specimen (Fig. 1a) and transplanted tissue (Figs. 1b-d) were representative of a high-grade astrocytoma, and included pleomorphic cells in a disorganized pattern, a few being multi-nucleated, frequent mitoses (mean = 3/high power field) pseudo-pallisading of tumour cells around areas of necrosis, and many thin-walled blood vessels showing endothelial proliferation. Electron microscopy of passaged tumour revealed fibrils (Fig. 2a) which are characteristic of glial cells (Raimondi, 1966). However, by the 9th passage these fibrils were no longer evident in the majority of the cells, and the predominant tumour-cell type had an irregular-shaped nucleus with a prominent nucleolus, with expanded rough endoplasmic reticulum. This type of cell (Fig. 2b) closely resembles that observed in *in vitro* cultures of astrocytoma by Guner *et al.* (1977). No virus-like particles have been observed in late-stage-passaged astrocytoma tissue by electron microscopical examination.

The karyotype of the tumour was checked at the 2nd, 5th and 7th passage and its human origin confirmed each time. The modal number of chromosomes was 47, with the extra chromosome being a metacentric, probably of Group B, which was present in all cells examined. A few polyploid cells were also seen.

*Effects of the immunological competence of host mice on growth of human gliomas*

For several serial transfers of the astrocytoma Grade IV, 4 pieces of tumour were implanted into each recipient. The mean take rate was 70%. We compared the observed frequency of animals having

FIG. 1.—(a) Diagnostic slide of surgical specimen of astrocytoma Grade IV, showing fibrillary astrocytes in a disorganised pattern with an example of pseudo-pallisading surrounding an area of necrosis. (b) The same tumour after 1st passage in immune-deprived mice, (c) the 6th passage with (d) the 10th passage. Sections stained with H & E; magnification × 415.

(a)

(b)

FIG. 2.—(a) Electron micrograph of transplanted astrocytoma (3rd passage) showing a typical astrocyte with lysosomes (L), mitochondria (M), irregular-shaped nucleus (N) and normal endoplasmic-reticulum (ER). Magnification × 10,000. Inset is detail from same cell demonstrating cytoplasmic fibrils (F). Magnification × 100,000. (b) Electron micrograph of same tumour (9th passage) showing loss of fibrils and the presence of expanded rough endoplasmic reticulum (ER). Magnification × 10,000.

TABLE II.—*Analysis of homogeneity of immune deprivation*

| Specimen | | No tumours/mouse 0 | 1 | 2 | 3 | 4 |
|---|---|---|---|---|---|---|
| Astrocytoma Grade IV | Observed frequency | 0 | 3 | 1 | 1 | 0 |
| 7th passage, takes = 8/20 | Expected frequency | 0·6 | 1·8 | 1·7 | 0·7 | 0·1 |
| Astrocytoma Grade III | Observed frequency | 0 | 0 | 2 | 0 | 3 |
| 1st passage, takes = 16/20 | Expected frequency | 0·01 | 0·1 | 0·8 | 2·0 | 2·0 |
| Astrocytoma Grade III | Observed frequency | 2 | 1 | 1 | 1 | 0 |
| Primary implants, takes = 6/20 | Expected frequency | 1·2 | 2·1 | 1·3 | 0·4 | 0·04 |

Expected tumour frequencies were obtained by binomial distribution analysis. Comparisons of observed and expected frequencies were made by Kolmogorov-Smirnoff analysis. $P > 0 \cdot 05$ in all cases.

1, 2, 3, 4 or no tumours with that expected on the basis that it was a random event. There was no significant difference between the observed and expected frequencies of tumour growth in 5 animals each implanted with 4 pieces of tumour in the 7th passage. Similarly, tumours arose in 5 mice implanted with an astrocytoma Grade III in the first passage with the expected frequency, as did tumours arising from a primary implant of astrocytoma Grade III. These results (Table II) indicate that our immune-deprived mice were probably homogeneous in relation to their capacity to support tumour growth, and that there was no interaction between tumours.

In a further series of experiments, the take rate of the astrocytoma Grade IV was determined in the congenitally athymic *nu/nu* (nude) mouse. These mice have been shown capable of supporting the growth of many different types of tumour (Rygaard and Povlsen, 1969; Giovanella *et al.*, 1974; Shimosato *et al.*, 1976). All tumours (1–4 pieces per mouse in its seventh passage) implanted into 7 nude mice grew. The growth curves of tumour in this type of animal, subjected to regression analyses, indicated that the mean tumour doubling time of 7·6 weeks was not significantly different ($P > 0{\cdot}1$) from the 7·4 weeks of the same tumour growing in immune-deprived mice (Fig. 3).

As a result of these studies, primary tumours have been implanted, where possible, to groups of both nude and immune-deprived mice. Of 7 tumours implanted

FIG. 3.—Growth of astrocytoma Grade IV (7th passage) in nude and immune-deprived mice. Each of 6 nude or immune-deprived mice were implanted with 1 piece of tumour in the subcutaneous space of the right flank and tumour volumes measured weekly.

(2 medulloblastomas, 1 astrocytoma Grade II, 3 astrocytomas Grade III and 1 meningioma) only 2 have grown (Table Ib). One, a medulloblastoma, was implanted in 2 nude (4 tumour pieces/mouse) and 5 immune-deprived mice (2 tumour pieces/mouse). One transplant grew in the nude mice (12·5% take rate) and one in each of 2 immune-deprived mice (20%

take rate). The second tumour that has grown in this experiment was an astrocytoma Grade III. One specimen has grown in 1 of 5 nude mice (4 tumour pieces/mouse) (5% take rate), whilst no tumours have grown so far in the corresponding immune-deprived mice. Thus, in this small number of specimens, primary human glioma tissue has not been shown to grow preferentially in nude mice compared with immune-deprived mice.

An alternative type of host has also been examined. During the preparation of immune-deprived mice, S. I. Detre (in preparation) in this laboratory has examined the effects of varying the time of thymectomy and the number of marrow cells given post-irradiation in an attempt to further deprive these animals. Groups of 5 mice were thymectomized by Detre at age 4 or 8 weeks and were reconstituted with either $5 \times 10^6$ or $10^5$ marrow cells post-irradiation. A further group of mice was thymectomized by us at age 5 weeks and reconstituted with $5 \times 10^6$ marrow cells. All animals were given implants of the astrocytoma Grade IV, 8th passage, one piece s.c. in each flank. Thymectomy at 8 weeks followed by whole-body irradiation and i.v. injection of $10^5$ marrow cells resulted in the highest tumour take rate of 100% (Table III). No difference

TABLE III.—*Effect of age at thymectomy and number of marrow cells on take rate of astrocytoma Grade IV*

| Age at thymectomy (weeks) | No. of marrow cells for reconstitution ($\times 10^5$) | Take rate (%) |
|---|---|---|
| 4 | 50 | 40 |
| 4 | 1 | 70 |
| 8 | 50 | 75 |
| 8 | 1 | 100 |
| 5 | 50 | 60 |

There were 5 mice in each group; all mice were age- and sex-matched cage-mates. They were sorted into groups after irradiation by applying random numbers. The marrow inoculum was in a final volume of 0·4 ml.

was found in tumour growth rate, nor in the interval between implantation and

TABLE IV.—*Comparison of take rates of astrocytoma Grade IV in various types of immune-deficient mice.*

| Tumour | Astrocytoma IV | |
|---|---|---|
| Standard immune-deprived mice | 69·2% | (36/52) |
| Modified immune-deprived mice | 100% | (10/10) |
| Nude mice | 100% | (24/24) |

Numbers in brackets are the number of tumours growing from the number of implants. Tumour was in its 7th and 8th passage.

perceptible tumour growth in the various types of immune-deprived mice. A comparison of the take rates of passaged astrocytoma Grade IV in nude, standard and modified immune-deprived mice is shown in Table IV.

From a total of 10 tumours, 7 primary specimens of astrocytoma Grade III have been implanted to these modified (thymectomy at 8 weeks, $10^5$ marrow cells post-irradiation) immune-deprived mice (Table Ic). One specimen implanted solely to 2 such animals, each given 2 pieces of tumour, has grown, with a 75% take rate. There was a delay in perceptible growth after implantation of 3 months. This tumour has been successfully transplanted to standard mice (thymectomy at 5 weeks, $5 \times 10^6$ marrow cells post-irradiation) (4 tumour pieces/mouse) where its take rate was 35% (tumour in first passage). Another astrocytoma Grade III has grown in both types of mice following primary implantation. A group of 5 standard and 5 modified immune-deprived mice were implanted with tumour, one piece in the subcutaneous flank tissue of each animal. The tumour take rate in the two groups was 40% and 20%, respectively. However, the delay in perceptible tumour growth in the standard immune-deprived mice was three months, whilst that in the modified immune-deprived mice was 6 months. Tumour from the former mice has been successfully passaged to further large groups (more than 30 mice per group) of standard immune-deprived mice where its take rate has increased to a mean of

85%. Thus, the initial low take rate has not been reflected in subsequent passages. One medulloblastoma, one ependymoma and one meningioma have also been implanted to the modified immune-deprived mice and have failed to grow. The meningioma also did not grow in a group of 5 nude mice. From the small number of primary implants in both types of immune-deprived mice, there does not appear to be any preferential take of tumour in the more immune-deprived animals.

### DISCUSSION

Our observations illustrate the feasibility of growing a proportion of human brain tumours in immune-deficient mice. At best, 50% of Grade III astrocytomas can be successfully implanted in such animals. The reason why the other 50% failed to grow are obscure. It could be due to residual immunological responsiveness in the mice, in which case nude or further-deprived animals might, in the long run, prove more receptive hosts: limited experience with such animals in the present study, however, does not support this view. Alternatively, it has been suggested (Cobb and Mitchley, 1974) that there is an imbalance between cell proliferation and death.

It is worth noting that 11/25 tumours, mostly high-grade astrocytomas from adults, grew in immune-deprived mice, compared with only 2/30 tumours from children. This may simply reflect the normal low incidence of high-grade astrocytomas in childhood. However, the failure of medulloblastomas from children to grow is surprising, in view of their known aggressive character in patients. Our preliminary attempts to grow these tumours in the brains of deprived mice were unsuccessful, giving no support to the notion that medulloblastomas may need a specific brain milieu for growth in a xenogeneic host.

The question of why transplants of highly malignant tumours often take several months to show perceptible growth is not easily answered. The fairly constant doubling times which were found, once growth had commenced, cannot be extrapolated back without some knowledge of cell-loss factors. It could be that relatively few tumour cells in the transplants are actually involved in subsequent growth. This is compatible with a doubling time of 2 months and a delay before palpability of 4 months or more. Further studies are required to resolve this issue.

Most, but not all, of the tumours which grew initially were transplantable into further hosts, which is the usual experience of those who have been involved with xenograft studies (Shimosato *et al.*, 1976; Berenbaum *et al.*, 1974). The growth rates and histopathological picture of the initial specimens were maintained in subsequent transplants, although future take rates improved in most instances. Again, no reason can be given for this effect, except to suppose that in some way the tumours had become conditioned to the physiological milieu of the mouse.

The eventual loss of fibrils in the majority of cells in the astrocytoma Grade IV, revealed by electron microscopy in later passages, may reflect a loss of differentiation, not detected by light microscopy. With time and increasing passage number in mice, the tumours will probably deviate more and more from their initial appearance and growth characteristics and may thereby become less suitable as models for chemotherapeutic studies.

The advantage of serially transplanting tumours, as opposed to primary implantation, in the most immunologically ineffective mice was revealed in the present study. The analysis of tumour takes per animal suggests that the residual immune response operating in standard immune-deprived mice may well prevent the growth of one tumour but be unable to prevent that of another in the same animals. That this is not due simply to total exhaustion of the immunological apparatus is suggested by the absence of interaction between transplanted tumours in the same animal.

The establishment and growth of human brain tumours in immunologically reduced mice is a feasible proposition. Although the growth of 2 human glioblastomas and 1 mengingioma in mice has been described previously from these laboratories and elsewhere (Cobb and Mitchley, 1974; Rana *et al.*, 1977) the present study deals with a larger series of the type of gliomas that can be expected to grow. Our preliminary observations suggest that such tumours in immune-reduced mice respond to chemotherapeutic agents (CCNU, BCNU) which have been successful in man. It is, as yet, unclear whether the experimental system we have described will be suitable for screening new agents of possible clinical therapeutic value, and for studies of the metabolism of human tumours.

The authors are grateful to Mr K. Till and Mr D. N. Grant of the Hospital for Sick Children, Mr L. S. Walsh, Mr A. E. Richardson and Mr D. Uttley of the Atkinson Morley's Hospital, and their respective theatre staffs, for kindly supplying the surgical specimens. We would also like to thank Miss S. Ludgate for her expertise in breeding the nude mice, Mrs P. Cartwright for her help with the electron microscopy and Mr J. Swansbury for assisting with the cytogenetical analysis.

### REFERENCES

AUSMAN, J. I., SHAPIRO, W. R. & RALL, D. P. (1970) Studies on the chemotherapy of experimental brain tumours: development of an experimental model. *Cancer Res.*, **30**, 2394.

BENDA, P., SOMEDA, K., MESSER, J. & SWEET, W. H. (1971) Morphological and immunochemical studies of rat glial tumours and clonal strains propagated in culture. *J. Neurosurg.*, **34**, 310.

BERENBAUM, M. C., SHEARD, C. E., REITTIE, J. R. & BUNDICK, R. V. (1974) The growth of human tumours in immunosuppressed mice and their response to chemotherapy. *Br. J. Cancer*, **30**, 13.

BLOOM, H. J. G. (1976) New therapeutic perspectives in brain tumours. In *I Tumori Infantili.* Eds P. Bucalossi, U. Veronesi, H. Emmanuelli & F. Fossati Bellani. Milan: Ambrosiana. p. 101.

COBB, L. M. & MITCHLEY, B. C. V. (1974) The growth of human tumours in immune-deprived mice. *Eur. J. Cancer*, **10**, 473.

GIOVENELLA, B. C., STEHLIN, J. S. & WILLIAMS, L. J. (1974) Heterotransplantation of human malignant tumours in nude thymus-less mice. *J. Nat. Cancer Inst.*, **52**, 921.

GUNER, M., FRESHNEY, R. I., MORGAN, D., FRESHNEY, M. G., THOMAS, D. G. T. & GRAHAM, D. I. (1977) Effects of dexamethasone and betamethazone on *in vitro* cultures from human astrocytoma. *Br. J. Cancer*, **35**, 439.

HANSTEEN, I. L. (1967) Chromosome studies in glial tumours. *Eur. J. Cancer*, **3**, 183.

LUBS, H. A. & SALMON, J. H. (1965) The chromosomal complement of human solid tumours. Part. II. Karotypes of glial tumours. *J. Neurosurg.*, **22**, 160.

MILLER, J. F. A. P., DOAK, S. M. A. & CROSS, A. M. (1963) Role of the thymus in recovery of the immune mechanism in the irradiated adult mouse. *Proc. Soc. Exp. Biol., Med.* **112**, 785.

RAIMONDI, A. J. (1966) Ultrastructure and the biology of human brain tumours *Prog. Neurol. Surg.*, **1**, 1.

RANA, M., PINKERTON, H., THORNTON, H. & NAGY, D. (1977) Heterotransplantation of human glioblastoma multiforme and meningioma to nude mice. *Proc. Soc. Exp. Biol. Med.*, **155**, 85.

ROSENBLUM, M. L., WHEELER, K. T., WILSON, C. B., BARKER, M. & KNEBEL, K. D. (1975) *In vitro* evaluation of *in vivo* brain tumour chemotherapy with 1,3-bis(2-chloroethyl)-1-nitrosourea. *Cancer Res.*, **35**, 1387.

RYGAARD, J. & POVLSEN, C. O. (1969) Heterotransplantation of a human malignant tumour to nude mice. *Acta Pathol. Microbiol. Scand.* (*A*), **77**, 758.

SHIMOSATO, Y., KANEYA, T. & NAGAI, K. (1976) Transplantation of human tumours in nude mice. *J. Nat. Cancer Inst.*, **56**, 1251.